# Non-contiguous finished genome sequence and description of *Paenibacillus gorillae* sp. nov.

**DOI:** 10.4056/sigs.5189179

**Published:** 2014-03-15

**Authors:** Mamadou Bhoye Keita, Roshan Padhmananabhan, Aurélia Caputo, Catherine Robert, Eric Delaporte, Didier Raoult, Pierre-Edouard Fournier, Fadi Bittar

**Affiliations:** 1 URMITE, Aix-Marseille Université, Faculté de médecine, Marseille, France; 2 IRD, University Montpellier 1, Montpellier, France; 3 King Fahad Medical Research Center, King Abdul Aziz University, Jeddah, Saudi Arabia

**Keywords:** *Paenibacillus gorillae*, genome, culturomics, taxonomo-genomics

## Abstract

Strain G1^T^ sp. nov. is the type strain of *Paenibacillus gorillae* a newly proposed species within the genus *Paenibacillus*. This strain, whose genome is described here, was isolated in France from the fecal sample of a wild western lowland gorilla from Cameroon. *P. gorillae* is a facultative anaerobic, Gram-negative, rod-shaped bacterium. Here we describe the features of this organism, together with the complete genome sequence and annotation. The 6,257,967 bp long genome (one chromosome but no plasmid) contains 5,856 protein-coding and 62 RNAs genes, including 60 tRNA genes.

## Introduction

Strain G1^T^ (= CSUR P205 = DSM 26181) is the type strain of *Paenibacillus gorillae* sp. nov. This bacterium is a Gram-negative, flagellated, facultative anaerobic, indole-negative bacillus that has rounded-ends. It was isolated from the stool sample of *Gorilla gorilla* subsp. *gorilla* as part of a culturomics study aiming at cultivating bacterial species found within gorilla feces. By applying large-scale culture conditions, culturomics has previously facilitated the isolation of many new bacterial species from human stool samples [1-3].

The genus *Paenibacillus* was created by Ash *et al*. about 20 years ago [4,5]. To date, this genus comprises 145 validly published species [6] of Gram-positive, Gram-negative or variable, mostly motile and spore-forming bacteria. Members of the genus *Paenibacillus* are ubiquitous bacteria isolated from various environments including soil, water, rhizosphere, food, insect larvae and normal human flora [7]. Moreover, *Paenibacillus* species were also isolated from or involved in human infections including wound infections, bacteremia and endocarditis [8-13]. Currently, a polyphasic approach that combines proteomics by MALDI-TOF spectral analysis, genomic data and phenotypic characterization is being used as a new approach to describe bacterial species [7,14-25].

Here we present a summary classification and a set of features for *P. gorillae* sp. nov. strain G1^T^ together with the description of the complete genome sequence and annotation. These characteristics support the circumscription of the species *P. gorillae* [26].

## Classification and features

**Figure 1:**
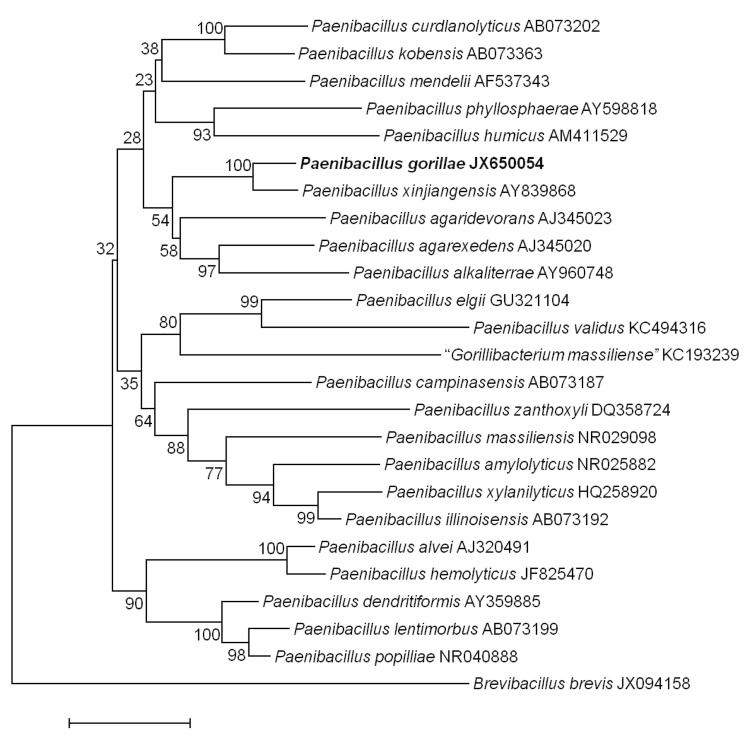
Phylogenetic tree highlighting the position of *Paenibacillus gorillae* strain G1^T^ relative to other type strains within the *Paenibacillus* genus. GenBank accession numbers are indicated in parentheses. Sequences were aligned using CLUSTAL X (V2), and phylogenetic inferences obtained using the maximum-likelihood method within the MEGA 5 software [41]. Numbers at the nodes are percentages of bootstrap values obtained by repeating the analysis 1,000 times to generate a majority consensus tree. *Brevibacillus brevis* was used as outgroup. The scale bar represents a 2% nucleotide sequence divergence.

In July 2011, a fecal sample was collected from a wild western lowland gorilla near Minton, a village in the south-central part of the DJA FAUNAL Park (Cameroon). The collection of the stool sample was approved by the Ministry of Scientific Research and Innovation of Cameroon. No experimentation was conducted on this gorilla. The fecal specimen was preserved at -80°C after collection and sent to Marseille. Strain G1^T^ (Table 1) was isolated in January 2012 by cultivation on Columbia agar with sheep blood 5% (BioMerieux, France). This strain exhibited a 98.28% 16S rRNA nucleotide sequence similarity with *Paenibacillus xinjiangensis*, the phylogenetically closest validly published *Paenibacillus* species (Figure 1). This value was lower than the 98.7% 16S rRNA gene sequence threshold recommended by Stackebrandt and Ebers to delineate a new species without carrying out DNA-DNA hybridization [42].

**Table 1:** Classification and general features of *Paenibacillus gorillae* strain G1^T^ according to the MIGS recommendations [27].

**MIGS ID**	**Property**	**Term**	**Evidence code^a^**
		Domain *Bacteria*	TAS [28]
		Phylum *Firmicutes*	TAS [29-31]
		Class *Bacilli*	TAS [32,33]
	Current classification	Order *Bacillales*	TAS [34,35]
		Family *Paenibacillaceae*	TAS [33,36]
		Genus *Paenibacillus*	TAS [4,5,37-39]
		Species *Paenibacillus gorillae*	IDA
		Type strain G1^T^	IDA
	Gram stain	negative	IDA
	Cell shape	rod-shaped	IDA
	Motility	motile	IDA
	Sporulation	Sporulating	IDA
	Temperature range	mesophilic	IDA
	Optimum temperature	25°C	IDA
MIGS-6.3	Salinity	growth in BHI medium + 2% NaCl	IDA
MIGS-22	Oxygen requirement	aerobic	IDA
	Carbon source	varied (see Table 2)	IDA
	Energy source	chemoorganoheterotrophic	IDA
MIGS-6	Habitat	gorilla gut	IDA
MIGS-15	Biotic relationship	free living	IDA
MIGS-14	Pathogenicity Biosafety level Isolation	unknown 2 gorilla feces	NAS NAS IDA
MIGS-4	Geographic location	Cameroon	IDA
MIGS-5	Sample collection time	July 2011	IDA
MIGS-4.1	Latitude	2.783938	IDA
MIGS-4.1	Longitude	13.030472	IDA
MIGS-4.3	Depth	surface	IDA
MIGS-4.4	Altitude	> 600 m above sea level	IDA

Evidence codes - IDA: Inferred from Direct Assay; TAS: Traceable Author Statement (i.e., a direct report exists in the literature); NAS: Non-traceable Author Statement (i.e., not directly observed for the living, isolated sample, but based on a generally accepted property for the species, or anecdotal evidence). These evidence codes are from the Gene Ontology project [40]. If the evidence is IDA, then the property was directly observed for a live isolate by one of the authors or an expert mentioned in the acknowledgements.

**Figure 2:**
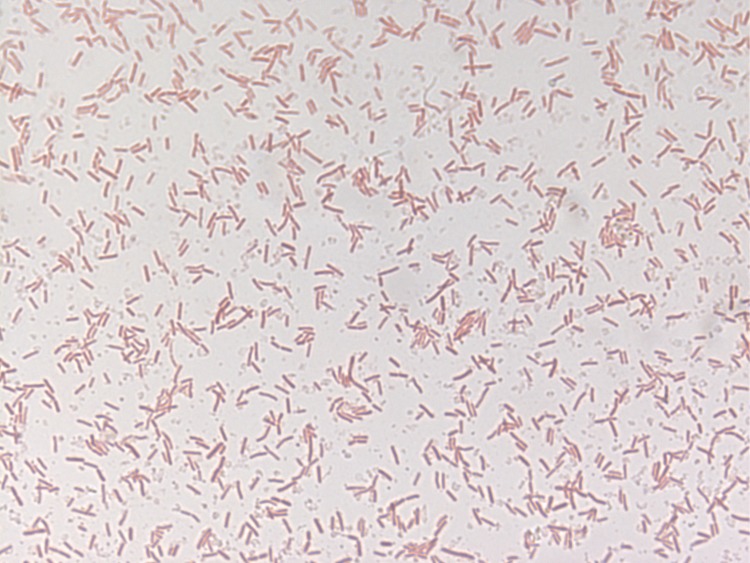
Gram staining of *P. gorillae* strain G1^T^

**Figure 3:**
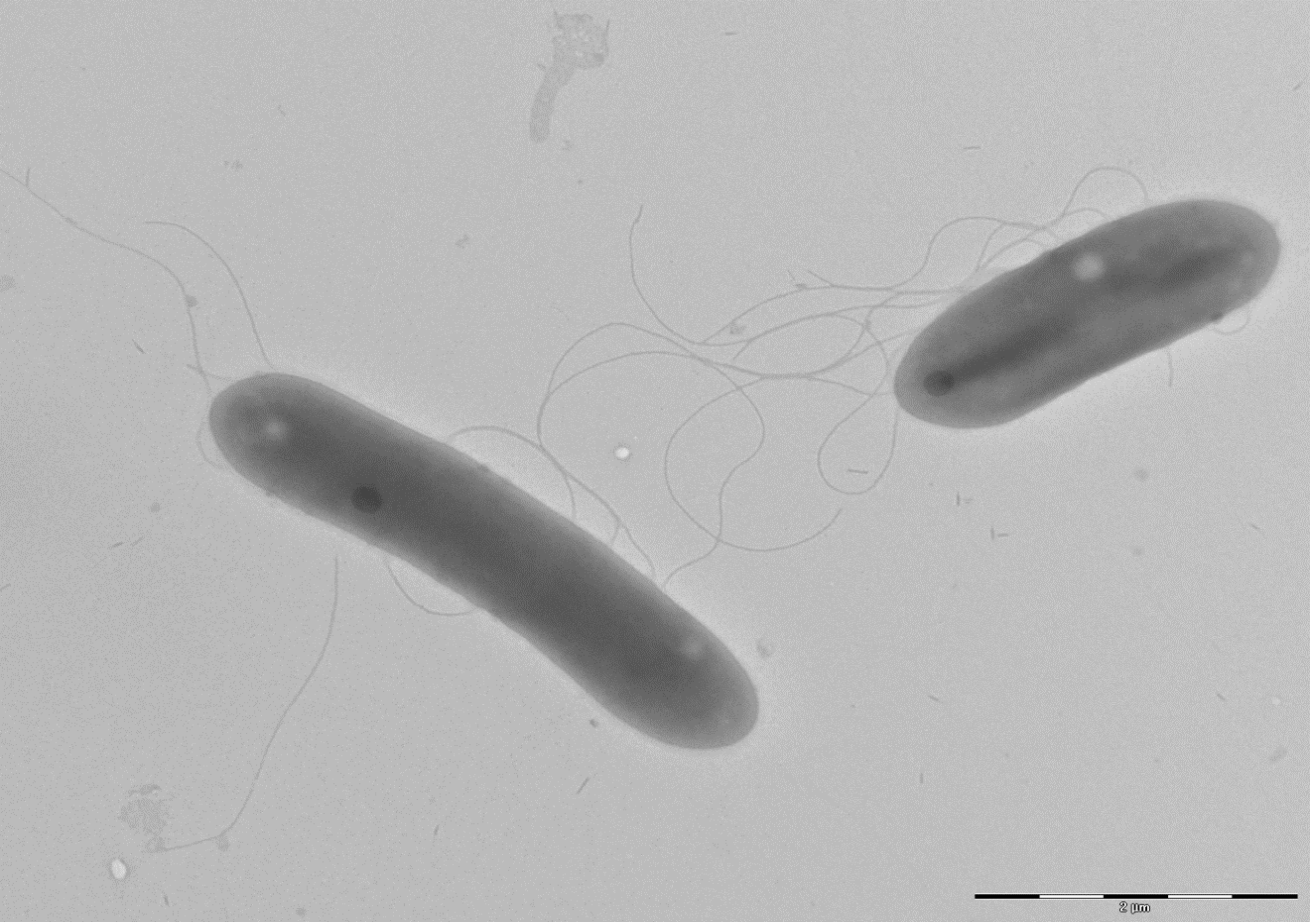
Transmission electron microscopy of *P. gorillae* strain G1^T^, using a Morgani 268D (Philips) at an operating voltage of 60kV. The scale bar represents 2 µm.

Different growth temperatures (25, 30, 37, 45°C) were tested. Growth occurred for the temperatures (25°C-37°C), but the optimal growth was observed at 25°C. Colonies were 2-8 mm in diameter on Columbia agar, appear whitish in color at 25°C and produce a clear liquid. Growth of the strain was tested under anaerobic and microaerophilic conditions using the GENbag anaer and GENbag microaer systems, respectively (BioMérieux), and in aerobic conditions, with or without 5% CO_2_. Growth was achieved under aerobic (with and without CO_2_), microaerophilic and anaerobic conditions. Gram staining showed Gram-negative bacilli (Figure 2). A motility test was positive. Cells grown on agar sporulate and the rods have a length ranging from 2.5 to 3.97 µm (mean 3.2 µm) and a diameter ranging from 0.76 to 0.83 µm (mean 0.79 µm) as determined by negative staining transmission electron microscopy (Figure 3).

Strain G1^T^ exhibited oxidase activity but not catalase activity. Using the API 50CH system (BioMerieux), a positive reaction was observed for D-mannose, amygdalin, L-arabinose, cellobiose, lactose, D-xylose, D-glucose, mannitol, arabinose, xylose, glycerol, D-galactose, N-acetylglucosamine, arbutin, aesculin, D-sorbitol, D-maltose, D-saccharose, D-trehalose, D-tagatose, L-rhamnose, salicin, adonitol, D-melibiose, D-raffinose, D-ribose, D-fructose and hydrolysis of starch. Negative reactions were observed for potassium gluconate, potassium 2-cetogluconate, inulin, D-melezitose, Glycogen, β-gentiobiose, D-turanose, methyl- αD-mannopyranoside and methyl- αD-glucopyranoside. Using the API ZYM system, negative reactions were observed for lipase (C14), a-chymotrypsin, esterase (C4), esterase lipase (C8), naphthyl-AS-BI-phosphohydrolase, phenylalanine arylamidase, leucine arylamidase, cystine arylamidase, valine arylamidase, glycine arylamidase, arginine arylamidase and β-glucosidase. Using the API Coryne system, positive reactions were observed for β-glucuronidase, alkaline phosphatase, α-glucosidase, α-galactosidase and N-acetyl-β-glucosaminidase activities. The urease reaction, nitrate reduction and indole production were negative. *P. gorillae* is susceptible to imipenem, rifampicin, gentamycin, nitrofurantoin and vancomycin, but resistant to metronidazole, trimethoprim/sulfamethoxazole, ceftriaxone, ciprofloxacin and amoxicillin.

When compared to other *Paenibacillus* species [43-46] and *Brevibacillus brevis* [47], *P. gorillae* sp. nov. strain G1^T^ exhibited the phenotypic differences detailed in Table 2.

**Table 2:** Differential phenotypic characteristics between *P. gorillae* sp. nov. strain G1^T^ and phylogenetically close *Paenibacillus* species

**Characteristic**	1	2	3	4	5	6
Gram stain	-	-	var	var	+	+/var
**Production of**						
Catalase	-	+	+	+	+	+
Oxidase	+	-	-	+	-	+
Nitrate reductase	-	+	+	-	+	+
Urease	-	+	+	na	na	-
Indole	-	-	+	+	na	-
**Utilization of**						
D-mannose	+	+	+	-	+	-
Amygdalin	+	-	-	-	+	-
L-Arabinose	+	w	-	-	+	-
Cellobiose	+	+	+	-	+	+
D-lactose	+	+	+	na	+	-
D-xylose	+	+	var	-	-	-
D-glucose	+	+	+	+	+	-
D-Mannitol	+	-	+	-	+	+
D-Arabinose	+	-	na	na	-	-
Glycerol	+	-	var	-	+	-
D-Galactose	+	-	+	na	+	+
Starch	+	+	+	+	+	-
N-acetylglucosamine	+	+	+	na	-	+
Arbutin	+	-	na	+	+	na
Aesculin	+	+	+	na	+	+
D-sorbitol	+	-	na	na	-	-
D-maltose	+	+	+	na	+	-
D-saccharose	+	+	na	-	+	-
D-trehalose	+	+	+	-	+	-
D-tagatose	+	-	na	na	-	-
Potassium gluconate	-	-	+	-	-	w
L-rhamnose	+	-	na	na	-	-
Salicin	+	+	-	-	+	-
Adonitol	+	-	na	+	-	-
D-melibiose	+	+	na	+	+	-
D-raffinose	+	+	na	+	+	-
D-ribose	+	-	+	na	+	-
D-fructose	+	+	w	na	+	-
**Habitat**	Gorilla gut	Gorilla gut	Roots of *Perilla frutescens*	Honeybee larvae	Human: blood culture	Environment: soil

Strain: 1, *Paenibacillus gorillae* G1^T^; 2, “*Gorillibacterium massiliense”* G5^T^; 3, *Paenibacillus elgii* SD17^T^; 4, *Paenibacillus alvei* BCRC 11220^T^; 5, *Paenibacillus massiliensis* 2301065 ^T^; 6, *Brevibacillus brevis* NBRC 15304^T^.

var: variable, +: positive result, -: negative result, na: data not available, w: weak positive result

**Figure 4:**
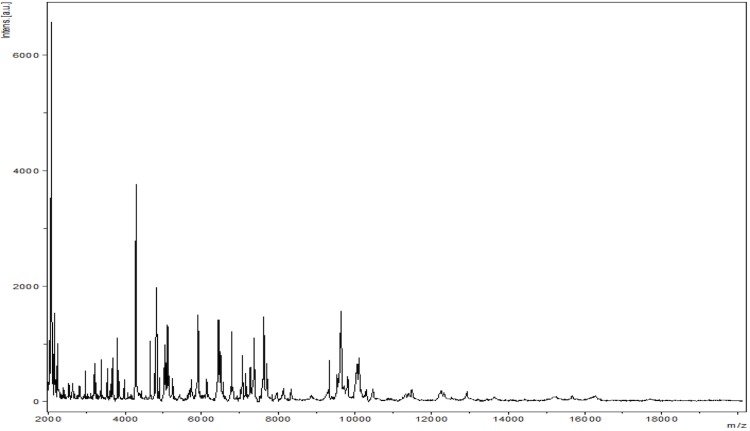
Reference mass spectrum from *P. gorillae* strain G1^T^. Spectra from 12 individual colonies were compared and a reference spectrum was generated.

**Figure 5:**
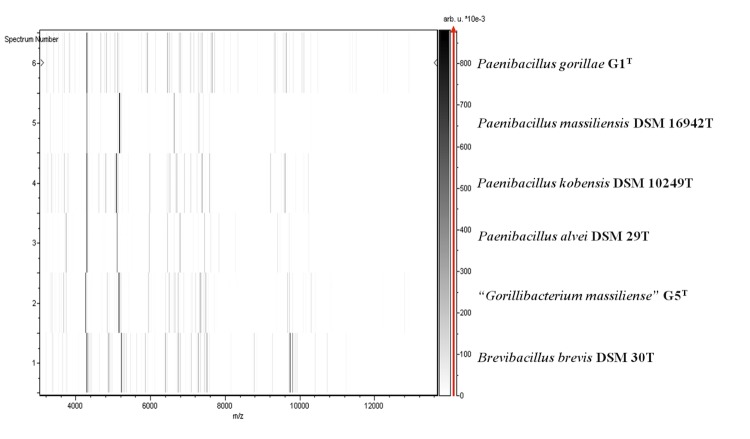
Gel view comparing *Paenibacillus gorillae* G1^T^ spectra with other members of the *Paenibacillus* genus (*P. massiliensis,* *P. kobensis,* and *P. alvei*) and with *Brevibacillus brevis* and “*Gorillibacterium massiliense”*. The Gel View displays the raw spectra of all loaded spectrum files arranged in a pseudo-gel like look. The x-axis records the m/z value. The left y-axis displays the running spectrum number originating from subsequent spectra loading. The peak intensity is expressed by a Gray scale scheme code. The color bar and the right y-axis indicate the relation between the color a peak is displayed with and the peak intensity in arbitrary units.

Matrix-assisted laser-desorption/ionization time-of-flight (MALDI-TOF) MS protein analysis was carried out as previously described [14] using a Microflex spectrometer (Bruker Daltonics, Leipzig, Germany). Twelve distinct deposits were made for strain G1^T^ from 12 isolated colonies. The 12 G1^T^ spectra were imported into the MALDI BioTyper software (version 2.0, Bruker) and analyzed by standard pattern matching (with default parameter settings) against 6,252 bacterial spectra including 123 spectra from 67 *Paenibacillus* species, used as reference data, in the BioTyper database. Interpretation of scores was as follows: a score > 2 to a validly published species enabled the identification at the species level, a score > 1.7 but < 2 enabled the identification at the genus level; and a score < 1.7 did not enable any identification. For strain G1^T^, the obtained scores ranged from 1.177 to 1.343, thus suggesting that our isolate was not a member of a known species. We incremented our database with the spectrum from strain G1^T^ (Figure 4). Spectrum differences with other of *Paenibacillus* species are shown in Figure 5.

## Genome sequencing information

### Genome project history

The organism was selected for sequencing on the basis of its phylogenetic position and 16S rRNA similarity to other members of the genus *Paenibacillus*, and is part of a “culturomics” study of the gorilla flora which aims to isolate all bacterial species within gorilla feces. It is the 44^th^ genome of a *Paenibacillus* species sequenced and the first genome of *Paenibacillus gorillae* sp. nov. sequenced. A summary of the project information is shown in Table 3. The Genbank accession number is CBVJ000000000 and consists of 167 contigs (150 large contigs). Table 3 shows the project information and its association with MIGS version 2.0 compliance [48].

**Table 3:** Project information

**MIGS ID**	**Property**	**Term**
MIGS-31	Finishing quality	High-quality draft
MIGS-28	Libraries used	454 paired-end 3- kb libraries
MIGS-29	Sequencing platform	454 GS FLX Titanium
MIGS-31.2	Sequencing coverage	17.2×
MIGS-30	Assemblers	Newbler version 2.5.3
MIGS-32	Gene calling method	Prodigal
	EMBL Date of Release	November 26, 2013
	EMBL ID	CBVJ000000000
MIGS-13	Project relevance	Study of the gorilla gut microbiome

### Growth conditions and DNA isolation

*P. gorillae* sp. nov. strain G1^T^, CSUR P205, DSM 26181, was grown aerobically on 5% sheep blood-enriched Columbia agar at 25°C. Four Petri dishes were spread and resuspended in 3×500µl of TE buffer and stored at 80°C. Then, 500µl of this suspension were thawed, centrifuged 3 minutes at 10,000 rpm and resuspended in 3×100µL of G2 buffer (EZ1 DNA Tissue kit, Qiagen). A first mechanical lysis was performed by glass powder on the Fastprep-24 device (Sample Preparation system, MP Biomedicals, USA) using 2×20 seconds cycles. DNA was then treated with 2.5µg/µL lysozyme (30 minutes at 37°C) and extracted using the BioRobot EZ1 Advanced XL (Qiagen). The DNA was then concentrated and purified using the Qiamp kit (Qiagen). The yield and the concentration was measured by the Quant-it Picogreen kit (Invitrogen) on the Genios Tecan fluorometer at 50ng/µl.

### Genome sequencing and assembly

A shotgun and a 3 kb paired end library were pyrosequenced on the 454_Roche_Titanium. This project was loaded on a 1/4 region for each application on PTP Picotiterplates. The shotgun library was constructed with 500ng of DNA as describes by the manufacturer Roche with the Rapid library Preparation kit for XL+. The concentration of the shotgun library was measured with a TBS fluorometer and determined to be 2.89E+09 molecules/µL. The paired-end library was prepared with 5 µg of bacterial DNA using the DNA fragmentation on a Covaris S-Series (S2) instrument (Woburn, Massachusetts, USA) with an enrichment size at 3.2kb. The DNA fragmentation was visualized with an Agilent 2100 BioAnalyzer on a DNA labchip 7500. The library was constructed according to the 454 GS FLX Titanium paired-end protocol (Roche). Circularization and nebulization were performed and generated a pattern with an optimum at 591bp. After PCR amplification through 17 cycles followed by double size selection, the single stranded paired-end library was quantified using the Quant-it Ribogreen kit (Invitrogen) on a Genios Tecan fluorometer at 691 pg/µL. The library concentration equivalence was calculated as 1.07E+10 molecules/µL. The library was stored at -20°C until further use.

The shotgun XL+ library was clonally amplified with 6 cpb in 2 emPCR reactions. The paired-end library was clonally amplified with 0.5 cpb in 3 emPCR reactions with the GS Titanium SV emPCR Kit (Lib-L) v2 (Roche). The yields of the emPCR were 16.9% and 8.61% respectively, and within the expected yield range of 5 to 20%, as recommended by the Roche procedure.

A total of 790,000 beads for each ¼ region per application were loaded on the GS Titanium PicoTiterPlate PTP Kit 70×75 and sequenced with the GS FLX Titanium Sequencing Kit XLR70 (Roche). The run was performed overnight and then analyzed on the cluster through the gsRunBrowser and Newbler assembler (Roche). A total of 339,189 passed filter wells were obtained and generated 108.46 Mb of sequences with a length average of 330 bp. The passed filter sequences were assembled using Newbler with 90% identity and 40-bp as overlap. The final assembly identified 11 scaffolds with 150 large contigs (>1.5kb) generating a genome size of 6.22 Mb corresponding to a genome coverage of 17.2×.

### Genome annotation

Open Reading Frames (ORFs) were predicted using Prodigal [49] with default parameters but the predicted ORFs were excluded if they spanned a sequencing gap region. The predicted bacterial protein sequences were searched against the GenBank database [50] and the Clusters of Orthologous Groups (COG) databases using BLASTP. The tRNAScanSE tool [51] was used to find tRNA genes, whereas ribosomal RNAs were found by using RNAmmer [52] and BLASTn against the GenBank database. ORFans were identified if their BLASTP *E*-value was lower than 1e-03 for alignment length greater than 80 amino acids. If alignment lengths were smaller than 80 amino acids, we used an *E*-value of 1e-05.

To estimate the mean level of nucleotide sequence similarity at the genome level between *P. gorillae* sp nov. strain G1^T^ and other *Paenibacillaceae* species, we use the Average Genomic Identity of orthologous gene Sequences (AGIOS) program. Briefly, this software combines the Proteinortho software [53] to detect orthologous proteins between genomes compared on a pair-wise basis, then retrieves the corresponding genes and determines the mean percentage of nucleotide sequence identity among orthologous ORFs using the Needleman-Wunsch global alignment algorithm.

## Genome properties

**Figure 6:**
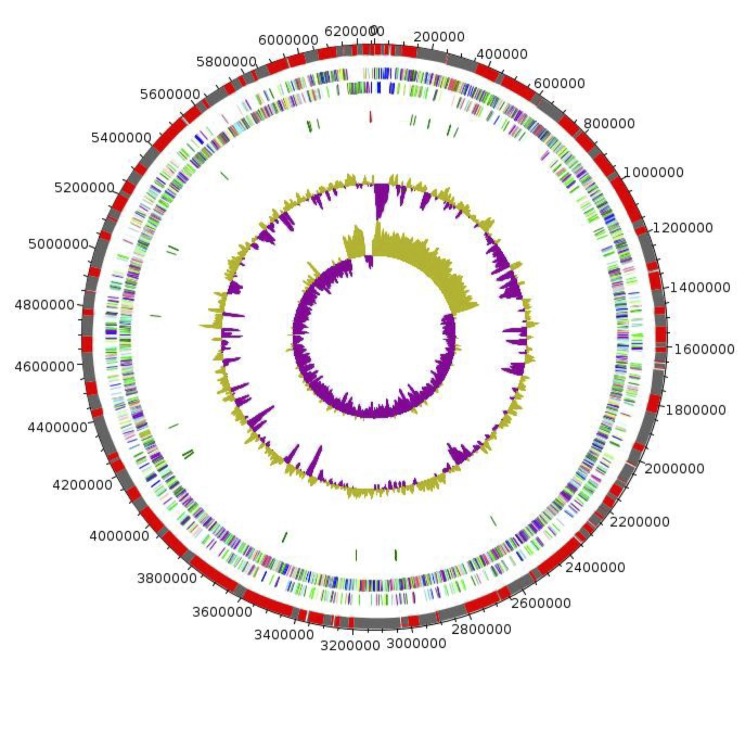
Graphical circular map of the chromosome. From outside to the center: Genes on the forward strand colored by COG categories (only genes assigned to COG), genes on the reverse strand colored by COG categories (only gene assigned to COG), RNA genes (tRNAs green, rRNAs red), G+C content and GC skew. Purple and olive indicating negative and positive values, respectively.

The genome 6,257,967 bp long (1 chromosome, but no plasmid) with a 48,80% G+C content (Figure 6 and Table 4). It is composed of 167 contigs (150 large contigs, 11 scaffolds). Of the 5,918 predicted genes, 5,856 were protein-coding genes and 62 were RNAs (1 gene is 16S rRNA, 1 gene is 23S rRNA and 60 are tRNA genes). A total of 4,296 genes (73.36%) were assigned a putative function (by COGS or by NR blast) and 304 genes were identified as ORFans (5.19%). The remaining genes were annotated as hypothetical proteins (917 genes, 15.66%). The distribution of genes into COGs functional categories is presented in Table 5. The properties and statistics of the genome are summarized in Tables 4 and 5.

**Table 4:** Nucleotide content and gene count levels of the genome

**Attribute**	Value	% of total^a^
Genome size (bp)	6,257,967	100
DNA G+C content (bp)	3,053,870	48.80
DNA coding region (bp)	5,416,322	86.55
Total genes	5,918	100
RNA genes	62	1.05
Protein-coding genes	5,856	98.95
Genes with function prediction	4,296	73.36
Genes assigned to COGs	4,305	73.51
Genes with peptide signals	809	13.81
Genes with transmembrane helices	1,440	24.59

^a^ The total is based on either the size of the genome in base pairs or the total number of protein coding genes in the annotated genome

**Table 5:** Number of genes associated with the 25 general COG functional categories

**Code**	**Value**	**%age**^a^	**Description**
J	195	3.33	Translation
A	0	0	RNA processing and modification
K	570	9.73	Transcription
L	186	3.18	Replication, recombination and repair
B	1	0.02	Chromatin structure and dynamics
D	31	0.53	Cell cycle control, mitosis and meiosis
Y	0	0	Nuclear structure
V	101	1.72	Defense mechanisms
T	354	6.05	Signal transduction mechanisms
M	247	4.22	Cell wall/membrane biogenesis
N	76	1.30	Cell motility
Z	2	0.03	Cytoskeleton
W	1	0.02	Extracellular structures
U	59	1.01	Intracellular trafficking and secretion
O	127	2.17	Posttranslational modification, protein turnover, chaperones
C	189	3.23	Energy production and conversion
G	591	10.09	Carbohydrate transport and metabolism
E	414	7.07	Amino acid transport and metabolism
F	96	1.64	Nucleotide transport and metabolism
H	125	2.13	Coenzyme transport and metabolism
I	144	2.46	Lipid transport and metabolism
P	365	6.23	Inorganic ion transport and metabolism
Q	147	2.51	Secondary metabolites biosynthesis, transport and catabolism
R	735	12.55	General function prediction only
S	339	5.79	Function unknown
-	1,551	26.49	Not in COGs

^a^ The total is based on the total number of protein coding genes in the annotated genome

## Genomic comparison of *P. gorillae* and other members of the family *Paenibacillaceae*.

Here, we compared the genome of *P. gorillae* strain G1^T^ with those of “*G. massiliense”* strain G5^T^, *P. elgii* strain B69, *P. alvei* strain DSM 29, *P. massiliensis* strain DSM 16942 and *B. brevis* strain NBRC 100599 (Table 6). The draft genome of *P. gorillae* is larger in size than that of “*G. massiliense”* (6.25 vs 5.54 Mb) and smaller in size than those of *P. elgii*, *P. alvei,* *P. massiliensis* and *B. brevis* (6.25 vs 7.96, 6.83, 6.39 and 6.3 Mb respectively). *P. gorillae* has a lower G+C content than those of “*G. massiliense”* and *P. elgii* (48.8% vs 50.39% and 52.6% respectively) but higher than those of *P. alvei* and *B. brevis* (48.8% vs 45.9% and 47.3% respectively) and slightly higher than *P. massiliensis* (48.8% vs 48.5%). The protein content of *P. gorillae* is lower than that of *P. elgii*, *P. alvei* and *B. brevis* (5,856 vs 7,597, 6,823 and 5,946 respectively) but higher than that of “*G. massiliense”* and *P. massiliensis* (5,856 vs 5,146 and 5,496 respectively) (Table 6). In addition, *P. gorillae* shares 1,987, 2,380, 2,055, 2,121 and 1,935 orthologous genes with “*G. massiliense”,* *P. elgii*, *P. alvei,* *P. massiliensis* and *B. brevis*, respectively (Table 7). The nucleotide sequence identity of orthologous genes ranges from 66.3 to 68.7% among previously published genomes, and from 65.7 to 68.6% between *P. gorillae* and the other studied genomes (Table 7), thus confirming its status as a new species. Table 7 summarizes the number of orthologous genes and the average percentage of nucleotide sequence identity between the different genomes studied.

**Table 6:** Genomic comparison of *P. gorillae* sp. nov., strain G1^T^ with four other members of the family *Paenibacillaceae***^†^**

**Species**	**Strain**	**NCBI accession number**	**Genome size (Mb)**	**G+C content**
*Paenibacillus gorillae*	G1^T^	CBVJ000000000	6.25	48.8
*“Gorillibacterium massiliense”*	G5^T^	CBQR000000000	5.54	50.39
*Paenibacillus elgii*	B69	AFHW00000000	7.96	52.6
*Paenibacillus alvei*	DSM 29	AMBZ00000000	6.83	45.9
*Paenibacillus massiliensis*	DSM 16942	ARIL00000000	6.39	48.5
*Brevibacillus brevis*	NBRC 100599	AP008955	6.3	47.3

**†**Species and strain names, GenBank genome accession numbers, sizes and G+C contents

**Table 7:** Genomic comparison of *P. gorillae* sp. nov., strain G1^T^ with four other members of the family *Paenibacillaceae*. †

	*P. gorillae*	*“G. massiliense"*	*P. elgii*	*P. alvei*	*P. massiliensis*	*B. brevis*
*P. gorillae*	**5,856**	67.8	68.6	68	67.8	65.7
*“G. massiliense”*	1,987	**5,146**	68.7	66.7	66.9	65.3
*P. elgii*	2,380	2,122	**7,597**	67.6	67	66.4
*P. alvei*	2,055	1,846	2,336	**6,823**	67.9	66
*P. massiliensis*	2,121	1,902	2,296	1,994	**5,496**	65.3
*B. brevis*	1,935	1,716	2,278	1,936	1,872	**5,946**

†numbers of orthologous proteins shared between genomes (lower left triangle), average percentage similarity of nucleotides corresponding to orthologous proteins shared between genomes (upper right triangle). Bold numbers indicate numbers of proteins per genome.

## Conclusion

On the basis of phenotypic (Table 2), phylogenetic and genomic analyses (taxonogenomics) (Table 7), we formally propose the creation of *Paenibacillus gorillae* sp. nov. that contains the strain G1^T^. This strain has been found in gorilla stool sample collected from Cameroon.

### Description of *Paenibacillus gorillae* sp. nov.

*Paenibacillus gorillae* (gor.il.lae, N.L. gen fem, of the gorilla from which the stool sample was obtained).

*P. gorillae* is a facultative aerobic Gram-negative. Optimal growth is achieved aerobically. A weak growth is observed in microaerophilic or anaerobic conditions. Growth occurs between 25 and 37°C, with optimal growth observed at 25°C. Cells stain Gram-negative, are rod-shaped, endospore-forming and motile with a mean diameter of 0.79 µm (range 0.76 to 0.83 µm) and a mean length of 3.2 µm (range 2.5 to 3.97 µm). Colonies are whitish and 2-8 mm in diameter on blood-enriched Columbia agar.

Catalase negative, oxidase positive. Using the API 50CH system (BioMerieux), a positive reaction is obtained for D-mannose, amygdalin, L-arabinose, cellobiose, lactose, D-xylose, D-glucose, mannitol, arabinose, xylose, glycerol, D-galactose, N-acetylglucosamine, arbutin, aesculin, D-sorbitol, D-maltose, D-saccharose, D-trehalose, D-tagatose, L-rhamnose, salicin, adonitol, D-melibiose, D-raffinose, D-ribose, D-fructose and hydrolysis of starch. Negative reactions are obtained for potassium gluconate, potassium 2-cetogluconate, inulin, D-melezitose, Glycogen, β-gentiobiose, D-turanose, Methyl- αD-mannopyranoside and Methyl- αD-glucopyranoside. Using the API ZYM system, negative reactions are obtained for lipase (C14), a-chymotrypsin, esterase (C4), esterase lipase (C8), naphthyl-AS-BI-phosphohydrolase, phenylalanine arylamidase, leucine arylamidase, cystine arylamidase, valine arylamidase, glycine arylamidase, arginine arylamidase and β-glucosidase. Using the API Coryne system, positive reactions are observed for β-glucuronidase, alkaline phosphatase, α-glucosidase, α-galactosidase and N-acetyl-β-glucosaminidase activities. The urease reaction, nitrate reduction and indole production were negative. *P. gorillae* is susceptible to imipenem, rifampicin, gentamycin, nitrofurantoin and vancomycin, but resistant to metronidazole, trimethoprim/sulfamethoxazole, ceftriaxone, ciprofloxacin and amoxicillin.

The G+C content of the genome is 48.8%. The 16S rRNA and genome sequences are deposited in GenBank under accession numbers JX650054 and CBVJ000000000, respectively. The type strain G1^T^ (= CSUR P205 = DSM 26181) was isolated from the fecal flora of a *Gorilla gorilla gorilla* from Cameroon.
